# Larval habitats of *Anopheles gambiae *s.s. (Diptera: Culicidae) influences vector competence to *Plasmodium falciparum *parasites

**DOI:** 10.1186/1475-2875-6-50

**Published:** 2007-04-30

**Authors:** Bernard A Okech, Louis C Gouagna, Guiyun Yan, John I Githure, John C Beier

**Affiliations:** 1 Centre for Biotechnology, Research and Development (CBRD), Kenya Medical Research Institute, P. O. Box 54840, Nairobi, Kenya; 2 Human Health Division, International Centre of Insect Physiology and Ecology (ICIPE), P. O. Box 30772, Nairobi, Kenya; 3 Département Société et Santé – UR 016, Institut de Recherche Pour le Développement (IRD), P.O. Box 64501, 34394 Montpellier Cedex 5, France; 4 Department of Epidemiology and Public Health, University of Miami School of Medicine, 12500 SW, 152^nd ^Street, Building B Miami, FL 33177, USA; 5 Program in Public Health, College of Health Sciences, University of California, Irvine, Hewitt Hall, Room 3038, Irvine, CA 92697-4050, USA; 6 Whitney Laboratory for Marine Bioscience, University of Florida, 9505 Ocean Shore Boulevard, St Augustine, 32080-8610, FL, USA

## Abstract

**Background:**

The origin of highly competent malaria vectors has been linked to productive larval habitats in the field, but there isn't solid quantitative or qualitative data to support it. To test this, the effect of larval habitat soil substrates on larval development time, pupation rates and vector competence of *Anopheles gambiae *to *Plasmodium falciparum *were examined.

**Methods:**

Soils were collected from active larval habitats with sandy and clay substrates from field sites and their total organic matter estimated. *An. gambiae *larvae were reared on these soil substrates and the larval development time and pupation rates monitored. The emerging adult mosquitoes were then artificially fed blood with infectious *P. falciparum *gametocytes from human volunteers and their midguts examined for oocyst infection after seven days. The wing sizes of the mosquitoes were also measured. The effect of autoclaving the soil substrates was also evaluated.

**Results:**

The total organic matter was significantly different between clay and sandy soils after autoclaving (P = 0.022). A generalized liner model (GLM) analysis identified habitat type (clay soil, sandy soil, or lake water) and autoclaving (that reduces presence of microbes) as significant factors affecting larval development time and oocyst infection intensities in adults. Autoclaving the soils resulted in the production of significantly smaller sized mosquitoes (P = 0.008). Autoclaving clay soils resulted in a significant reduction in *Plasmodium falciparum *oocyst intensities (P = 0.041) in clay soils (unautoclaved clay soils (4.28 ± 0.18 oocysts/midgut; autoclaved clay soils = 1.17 ± 0.55 oocysts/midgut) although no difference (P = 0.480) in infection rates was observed between clay soils (10.4%), sandy soils (5.3%) or lake water (7.9%).

**Conclusion:**

This study suggests an important nutritional role for organic matter and microbial fauna on mosquito fitness and vector competence. It shows that the quality of natural aquatic habitats of mosquito larvae may influence malaria parasite transmission potential by *An. gambiae*. This information can be important in targeting larval habitats for malaria control.

## Background

Malarial vectors in the *Anopheles gambiae *complex are known to use diverse small water bodies as larval habitats [1]. These habitats differ in physical as well as biological characteristics, which directly influence the distribution and abundance of larval mosquito populations in nature [2]. While it is known from laboratory studies that larval mosquito nutrition affects vector competence [3,4], the factors that determine adult *An. gambiae *fitness for malaria parasite transmission in the field are unclear, with only anecdotal evidence suggesting a role for larval habitat productivity [4].

The presence of *An. gambiae *larvae in small water bodies has been associated with biotic characteristics such as plankton, suggesting a contribution by plankton to the growth and development of the larvae in the field [5,6]. It has been shown that nutritional resources in larval habitats determine adult mosquito size [3], and that a relationship exists between size and parasite infectivity [4], yet no studies have been conducted to determine if natural mosquito larval habitat substrates have an effect on mosquito productivity or *Plasmodium falciparum *parasite infectivity in the adult mosquitoes. The underlying influences of soil type and organic matter content on larval development, adult mosquito productivity and on the corresponding malaria parasite transmission potential of *An. gambiae *have not been given much attention.

To answer these questions the effect of different soil substrates on larval development and adult vector competence of *An. gambiae *for *P falciparum *parasites was evaluated. Soil substrates were sampled from larvae inhabited water bodies from two geographically isolated field sites in western Kenya. By using *P. falciparum *gametocytes from human volunteers and *An. gambiae *reared in water with natural larval soil substrates, the natural process of *P. falciparum *development in mosquitoes was mimicked and thus more light shed on regulatory mechanisms that have not been well characterized in nature. This study will enhance the understanding of the nutritional value of larval mosquito aquatic habitats and their potential influence on vector competence. Such information may prove useful for developing malaria control strategies that target larval habitats.

## Materials and methods

### Study area

The study was conducted at the International Centre of Insect Physiology and Ecology (ICIPE) Thomas Odhiambo Campus located in south-western Kenya on the shores of Lake Victoria, in the Suba District. This area is surrounded by hills on the south and southeast with the rest of the area opening up into the low-lying basin facing the shores of lake. Within the lake itself are several islands; the nearest is Rusinga, which is joined to the mainland by a causeway. Stagnant water bodies that make potential mosquito breeding sites are found on the shores of the lake. The mosquito breeding habitats in the study area are diverse. They include small pools, hoof prints, drains, ditches, river edges, ponds, marshes, man-made holes, and peri-domestic cemented water containers [7]. The mean annual rainfall in this area ranges from 1200–1600 mm per year, but rainfall varies by season and year (ICIPE Thomas Odhiambo Campus meteorological station data). The long rains usually run from March to June and the short rains run from October to December. Malaria transmission in this area is endemic, with *An. gambiae *s.s., *An. arabiensis*, and *An. funestus *contributing and sustaining malaria transmission levels estimated at between 0 and 1.55 infectious bites per person per month [8].

The two geographically distinct sites used in this study were identified based on abundance of larvae, differences in soil type, and vegetation cover. The first site (Lat: 0°, 28.26' S; Long: 34°, 17.311' E) near Lwanda Village is a peninsula in a marshy area with predominantly black cotton soil (clay soil). The site is grazed by many herbivores (cows, donkeys, sheep, goats, and hippopotami) and is therefore littered with animal dung and hoof print ponds. The second site (Lat: 0° 24' S; Long: 34° 10' E) near Wasaria Village on Rusinga Island is a well-drained area with sandy soils that spread over a wide area and wash into the lake. This area has scattered patches of grass where herbivores graze (cows, goats and sheep) and fishermen mend nets and sort their fish. In addition to animal dung and hoof print ponds, fish debris is abundant in the mosquito aquatic habitats in this area.

### Selection and sampling of habitat soils for experiments

To determine which larval habitats to use, the two sites described above were surveyed to identify productive habitats. A visual inspection of the water bodies was done and those observed with anopheline larvae were selected. The densities of larvae in the water bodies were estimated. In small habitats like hoof print pools, larval densities were estimated by evacuating the water using a Pasteur pipette to recover the larvae after which they were counted. Two larval habitats were selected in each of the study sites (Lwanda and Rusinga) based on high larval density. Soils were collected from these habitats at the end of the each rainy season by digging out the top 10 cm and transporting the soil to the ICIPE Thomas Odhiambo field campus, where we set up experimental larval habitats. The experimental larval habitats were set up under semi-field conditions inside modified greenhouses [9] to overcome the many uncontrollable factors in natural conditions, such as rainfall, other weather conditions, security of sites, co-habiting predatory organisms that might affect vector productivity, and impacts of animals seeking water. The semi-field set up allowed us to test many soil samples and to reliably produce mosquitoes for fitness measurements and vector competence tests.

### Manipulation of soils collected from field sites

The soils were kept in a screen house at the ICIPE-Thomas Odhiambo campus for approximately two weeks to allow them to dry out before being used in experiments. This ensured that any mosquito eggs collected with the soils would dry up and be rendered non-viable [10,11]. Flooding and exposure to water to hatch any eggs would have altered the biotic and physico-chemical composition of the soils and was avoided. The larger particles were broken into finer pieces and 1,000 cm^3 ^of the soil measured and spread evenly on the bottom of rearing troughs to make substrates.

Over a four month period, the sites were visited and soils collected. A total of six visits were made and after each visit, the organic matter content of soils was determined by weighing 10 grams of each soil type that had been ground and dried and then burning it over a Bunsen burner at a temperature of 150°C in an open crucible for three hours. This process converted organic matter to water and carbon dioxide. The remaining soil material was then weighed with a micro scale balance. The difference in the two weights represented the percent loss due to burning, which directly reflected the total organic matter in the soil sample. These soil samples were however not used for rearing mosquitoes. The effect of soil microbiota was assessed by autoclaving soil samples at 120°C for 30 minutes to kill microbes and inhibit their activity. Another soil sample was simply flooded without sterilization.

### Rearing mosquitoes on soil substrates

Gravid colony-reared *An. gambiae *s.s. mosquitoes (MBITA strain) were allowed to lay eggs; the eggs were later dispensed into plastic boats and allowed to hatch. Twenty four hours after hatching, 100 first instar larvae were dispensed into plastic troughs lined with unautoclaved and autoclaved clay and sandy soil. In each of these experiments, a control experiment was set up with lake water only. The soil substrates were autoclaved to reduce microbial activity. The first instar larvae were introduced into equal sized pans (30 × 30 × 5 cm) and their development monitored. All the troughs were exposed to ambient climate conditions in the modified greenhouse. No extra food was given to the developing larvae in the experiments except in the lake water set up. The time for larvae to develop into pupae (larval development time) and the number of pupae forming (pupation rate) was recorded for each rearing pan. Pupae were collected, counted, and transferred to small cages (15 × 15 × 15 cm) where the number of emerging adults (emergence rate) was counted. The adults were provided access to 10% glucose solution and kept in cages for 3–5 days before being used in experimental infection studies with *P. falciparum *parasites.

### Recruitment of *P. falciparum *gametocyte carriers

The procedure for recruiting *P. falciparum *gametocyte positive human volunteers for experimental infection studies has been described in detail elsewhere [12]. Briefly, gametocytes were obtained from human volunteers recruited from among patients attending the outpatient department of the Mbita Health Centre and from villagers and school-age children from the community surrounding the ICIPE Thomas Odhiambo Campus. Only gametocyte carriers within the age group of 3–30 years were recruited, in accordance with the guidelines of the ethical review boards of the Kenya Medical Research Institute (KEMRI) and the University of Miami, USA.

Thick and thin blood smears were collected on slides, and standard parasitological techniques were employed to identify *P. falciparum *asexual and/or sexual parasites. Only volunteers with observed gametocyte densities greater than 16 gametocytes per microliter of blood were selected and used in the study. The goals of the study were explained to these individuals in a language they could understand, and individuals over 18 years of age were requested to sign an informed consent form; if volunteers were under 18 years, their assent was requested and their parents or guardians signed the informed consent form on their behalf.

### Experimental infection and assessment of oocyst stages in mosquitoes

Three to five days after emerging from the soil substrates, batches of 50–100 female mosquitoes were put in paper cups, labeled according to the type of habitat soil substrate, and starved for six hours prior to experimental blood feeding via Parafilm^® ^membrane. The blood for this experimental infection was obtained by a clinician from the Mbita Health Centre, who withdrew 2 ml of venous blood into heparinized tubes from each recruited gametocyte carrier. This blood sample was immediately offered to mosquitoes in pre-warmed (37°C) artificial membrane mini-glass feeders. Mosquitoes were allowed to feed for 15 minutes, after which unfed mosquitoes were removed. The engorged mosquitoes were held at 27°C and 70% relative humidity. To detect oocysts in the midgut, mosquito midguts were dissected on day 7 post infection, then stained with 2% mercurochrome, and each one examined under a microscope at a magnification of 10×. The wings of the dissected mosquitoes were mounted on slides and measured to determine the size of the mosquito [13].

### Statistical analysis

The independent sample T-test (assuming inequality of variances) was used to determine differences in the total organic matter between unautoclaved and autoclaved clay and sandy soils. A generalized linear model (GLM) multivariate analysis was used to determine the effects of habitat type and autoclaved soils on larval development time, number of pupae formed, and number of adults emerging. GLM univariate analysis was used to test the effect of the two factors (soil type, autoclaving) on oocyst intensities in mosquito midguts. The differences in larval development time and oocyst intensities were explored further by Least Significant Difference (LSD) in GLM multivariate and univariate analysis, respectively, for the different experimental groups. Pearson's chi square test was applied to examine the relationships among pupation rates, emergence rates, and soil types. The differences in the mean oocyst intensities between groups were analysed using ANOVA and the Tukey test.

## Results

### The organic content in unautoclaved and autoclaved soils

A total of 6 samples of the soils collected were tested for organic matter content. There were no significant differences in the total organic matter between unautoclaved clay and unautoclaved sandy soils (independent sample T-test: P = 0.853). In autoclaved soils, a significant difference was seen in total organic matter content between clay and sandy soils (T-test: P = 0.022), with more organic matter observed in clay soils (2.12% ± 0.09) than in sandy soils (1.07% ± 0.39). The process of autoclaving removes microbes that degrade organic matter, hence a higher organic matter content was observed after autoclaving.

### Effect of larval habitat soils on larval development

The larval development time to pupae in clay soils (5.0 days ± 0.57) and sandy soils (5.7 days ± 0.88) were shorter compared to mosquito larvae reared in lake water (6.33 days ± 0.33). This trend was similar in autoclaved soils (Table 1), suggesting that autoclaving (that reduced soil microbial activity) did not affect larval development time. However, the larval development time were not significantly different before (P = 0.606) or after autoclaving (P = 0.661) although a generalized linear model (GLM) multivariate analysis identified habitat type as a significant factor affecting larval development into pupae, with the replicate as a significant covariant (Table 2). The interaction between habitat type and replicate suggests that temporal changes in the habitat soil composition might affect larval development parameters. The numbers of pupae forming and emerging adults were not significantly affected by habitat type or soil autoclaving (Table 2). Between un-autoclaved soil types, mosquitoes sizes (clay soils = 3.134 ± 0.26; and sandy soils = 3.131 ± 0.26) were not significantly different (ANOVA: df = 1, F = 0.010, P = 0.920). However, when the soils were autoclaved, the sizes of mosquitoes were significantly different (ANOVA: df = 1, F = 7.161, P = 0.008) between clay (3.012 ± 0.205) and sandy soils (2.946 ± 0.28). On the other hand, within soil types, autoclaving did not significantly affect the size of mosquitoes produced in clay soils (ANOVA: df = 2, F = 0.233, P = 0.796) or in sandy soils (ANOVA: df = 2, F = 0.120, P = 0.888).

**Table 1:** The influence of autoclaving larval habitat soils on fitness parameters of *An. gambiae *mosquitoes.

	Rearing substrates for larvae			
	
Mosquito parameter measured.	Insectary	Clay soils	Sandy soils	Prob.
				
*Un-autoclaved soils*				
Larval development time (d) ± SD	6.33 ± 0.33	5.0 ± 0.57	5.7 ± 0.88	0.606
Pupation rates	94.1%	43.3%	55.3%	0.260
Wing sizes ± SE	3.12 ± 0.22	3.08 ± 0.10	3.16 ± 0.07	0.523
*Autoclaved soils*				
Larval development time (d) ± SD	6.3 ± 0.33	5.7 ± 0.88	5.3 ± 0.88	0.661
Pupation rates	82.7%	55.3%	65.7%	0.451
Wing size ± SE	2.85 ± 0.12	2.94 ± 0.11	2.86 ± 0.13	0.641

#Values are means ± SD of readings from 12 experiments for all the 3 habitat types. In the control group, mosquitoes were reared in lake water using standard protocols.

**Table 2:** A GLM multivariate analysis of variance test on the effect of habitat type and autoclaving on *An. gambiae *mosquito larval development.

Source of variation	DF	Days to pupation^¶^		No. of pupae forming^δ^		No of emerging adults^€^	
				
		F	Prob.	F	Prob.	F.	Prob.
							
Intercept	1	186.78	>0.001	34.78	>0.001	32.99	>0.001
Site	2	11.65	0.001*	1.67	0.224	0.98	0.400
Treatment	1	0.000	0.998	0.000	0.998	0.000	0.998
Replicate	1	83.51	>0.001*	1.032	0.327	1.716	0.211
Site × Replicate	2	5.559	0.017*	0.647	0.539	0.428	0.660
Site × Treatment × Replicate	3	0.000	0.999	0.000	0.999	0.000	0.999
Error	14						

* P < 0.05.

^¶ ^the time for larvae to develop to pupae.

^δ ^the number of pupae forming from a given number of larvae.

^€ ^the number of adults emerging from a given number of pupae.

### Effect of larval habitat soils on oocyst infections in mosquitoes

**Figure 1:**
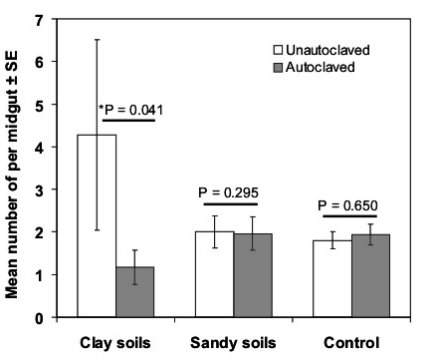
The effect of autoclaving soils from clay and sandy larval habitats on the oocyst infection intensities in the midguts of *An. gambiae *mosquitoes.

The oocyst infection rates in mosquitoes reared in clay soils was (10.4%) slightly higher than in mosquitoes reared in sandy soils (5.3%) or in control (7.9%) (Table 3). No significant differences in oocyst infection rates between *An. gambiae *mosquitoes reared in un-autoclaved soils were observed (Chi square: χ^2 ^= 27.71, df = 28, P = 0.480). A GLM univariate analysis showed that habitat type and autoclaving soils significantly affected oocyst intensities in mosquito midguts (Table 4). As expected, the gametocyte density and mosquito size were significant factors affecting oocyst intensities (Table 4). The reduction in oocyst infection intensities (Figure 1) was significant in autoclaved clay soils (Tukey Test: P = 0.041) but not on autoclaved sandy soils (Tukey Test: P = 0.295).

**Table 3:** The infectivity of *An. gambiae *s.l. mosquitoes reared on unautoclaved clay and sandy soil substrates obtained from mosquito larval habitats in western Kenya and then co-infected with different gametocyte carriers (mean gametocyte density = 173.8 ± 121.4).

Gametocyte		Oocyst infection rates			Oocyst intensity		
		
Carrier #	Density	Lab	Clay	Sandy	Lab	Clay	Sandy
							
1	48	15.4 (2/13)	21.6 (8/37)	0 (0/56)	2.0	2.1	0
2	16	2.2 (1/45)	9.5 (8/84)	0 (0/38)	2.0	1.6	0
3	32	9.4 (5/53)	27.3 (12/44)	0 (0/51)	1.4	2.0	0
4	32	ND	8.7 (2/23)	0 (0/34)	ND	2.5	0
5	128	40 (2/5)	0 (0/20)	13.9 (16/115)	2.0	0	2.0
6	16	9.7 (3/31)	0 (0/17)	2.8 (3/108)	2.33	0	1.7
7	16	6.9 (2/29)	0 (0/40)	16.1 (5/31)	1.50	0	2.8
8	16	0 (0/14)	0 (0/23)	0 (0/24)	0.0	0	0.0
							
		7.9% (15/190)	10.4% (30/288)	5.3% (24/457)	1.60 ± 0.29	2.06 ± 0.18	1.62 ± 0.59

Only 8 out of 22 experiments successfully yielded infected mosquitoes. The analysis of mosquito infection was done individually to account for differences in infectiousness among gametocyte carriers and ONLY successful infection experiments have been used in this table.

**Table 4:** A GLM univariate test on the effect of habitat type and treatment of habitat soils by autoclaving on midgut oocyst intensities in *An. gambiae *mosquitoes.

Source of variation	DF	MS	F	Prob.
				
Intercept	1	1.275	6.423	0.022
^†^Habitat type	1	0.978	4.928	0.041*
^$^Treatment	1	1.214	6.120	0.025*
Habitat type × Treatment	1	0.664	3.347	0.086
Total organic matter	1	0.401	2.018	0.175
Gametocyte density	1	1.580	7.965	0.012*
Mosquito size	1	1.193	6.011	0.026*
Error	16	0.198		

^†^Habitat refers to clay or sandy soils mixed with water. ^$^Treatment refers to autoclaving or not autoclaving the soils; *Probability, P < 0.05.

## Discussion

This study has demonstrated that productive mosquito aquatic habitats as detected by soil organic matter content and microbial activity, contributes to fitness and vector competence of *An. gambiae*, which is the major *P. falciparum *malaria transmitter in sub-Saharan Africa. These two soil parameters may directly contribute to the variations in malaria parasite transmission seen with this vector mosquito species in western Kenya [8]. The results of this study have demonstrated that higher organic matter and more microbial activity increases mosquito size and parasite infectivity. The depletion of microbial activity by autoclaving the soils resulted in reduced food resources and reduced fitness in the adult mosquitoes. Furthermore, the reduced oocyst intensities in the midgut of *An. gambiae *mosquitoes reared in autoclaved soils (devoid of microbes) suggest the influence of soil microbes on the vector competence of the malaria vector *An. gambiae*. Although these results give us an indication about the influence of habitat soil substrates, several experimental factors call for caution when interpreting these results. These factors are the exclusion of some infection experiments due to low mosquito numbers used, zero infected mosquitoes (Table 3), or the variable number of mosquitoes used in experiments (10–30 mosquitoes per group). However, the difference observed from the effect of soils was independent of the number of mosquitoes included in the analysis.

Smaller sized mosquitoes emerging from autoclaved soils suggests a clear role for soil microbes on larval mosquito nutrition and development; this is consistent with previous reports [14,15]. This study suggests that clay soils are more biologically active, having more nutritive value in the form of organic matter for the growth and development of mosquitoes. Soil microbiota is responsible for decomposing organic matter, and their removal through autoclaving means that the available organic matter in the soils is not degraded. The products of the organic matter breakdown increase the amount of organic molecules that contributes to larval nourishment for growth and survival [16]. Although soil quality and the corresponding nutritive value may vary between natural breeding sites of *An. gambiae*, the lack of major differences in the larval development time, number of pupae forming, or number of emerging adults between unautoclaved and autoclaved soils suggest that the larvae of *An. gambiae *can develop well on non-microbial components of soils. Furthermore, it shows that larvae of *An. gambiae *are adaptable to conditions of reduced nutritional quality in their natural habitats. Considering the diversity of water bodies being utilized by *Anopheles *larvae in nature [1], it is possible that larvae may survive on dissolved organic and inorganic molecules [6,16]. The results herein provide important clues of other potential food resources available for *Anopheles *mosquito larvae in natural water bodies.

The response of *An. gambiae *mosquitoes to malaria parasite infection varies as a function of habitat type, biological activity of the habitat, size of the mosquito, and gametocyte density. This study determined that soil organic matter content was different between habitat types and may have directly influenced the sizes of the mosquitoes produced. Although other factors, such as temperature [17] or larval densities of larvae in a habitat [17], may also affect the size of mosquitoes produced, these were controlled for in the experimental setup and therefore did not contribute to the differences observed. The size of the mosquito may influence oocyst intensities in the midgut of naturally infected mosquitoes [4]. The observed high organic matter content in clay soils, may have led to the production of larger mosquitoes. This in turn led to higher oocyst intensities in mosquitoes reared in predominantly clay habitats. Water bodies on clay soils appear turbid because of the presence of particulate matter in colloidal suspension. To quantify the amount or identity of the particulate matter in clay habitats was not logistically possible in this study. However, *Anopheles *mosquito larvae may feed through their suspension feeding mechanism on such particles to derive extra nutriment [16]. This extra nutriment could have led to the higher fitness observed in emerging adult mosquitoes on clay soils.

The influence of natural larval habitats on *An. gambiae *fitness and vector competence should be carefully considered to understand the ecology of this important malaria vector. Furthermore the effect of the factors identified in this study, as well as other unknown or uncontrolled factors in larval aquatic habitats, may be part of the system that determines the heterogeneity of larval developmental stages in water bodies and adult vector mosquito production in nature. In urban environments, it is assumed that environmental pollutants are likely to reduce mosquito fitness (Paul Miregi, pers. comm.) and vector competence. As demonstrated in this study, changes in habitat quality due to microbial transformation or in the organic matter content may directly govern the distribution of *Anopheles *mosquitoes and the risk of malaria transmission in endemic areas. Inherently, it is of value to identify such productive larval habitats in nature as their removal will greatly contribute to malaria control efforts.

## Authors' contributions

BAO – Designed experiments, collected and analyzed data, wrote manuscript; LCG – Data collection and review of manuscript; JCB – Review of data and manuscript; GY – Review of manuscript; JIG – Administrative support and review of manuscript. All authors have read and agree with the contents in the paper.
